# Equine (*Equus caballus*) primary post-natal brain-derived cells: immortalization and preliminary reprogramming attempts to induced pluripotent stem cells

**DOI:** 10.3389/fvets.2026.1931938

**Published:** 2026-10-07

**Authors:** Antonella Molinari, Camilla Lucca, Stefania Vogiatzis, Valentina Moccia, Massimiliano Babbucci, Luca Peruzza, Enrico Negrisolo, Marta Trevisan, Luisa Barzon, Cinzia Centelleghe, Sandro Mazzariol, Valentina Elena Giuditta Zappulli

**Affiliations:** 1 Department of Comparative Biomedicine and Food Science, University of Padua, Padua, Italy; 2 Department of Molecular Medicine, University of Padua, Padua, Italy; 3 Department of Agronomy, Food, Natural Resources, Animals, and Environment, University of Padua, Padua, Italy

**Keywords:** brain, brain organoids, equine, immortalized cells, induced pluripotent stem cells, *One Health*, primary cells

## Abstract

Equines represent an underexplored yet highly relevant model for studying neurological diseases within a *One Health* framework. Here, we report the first establishment of post-natal equine brain-derived *in vitro* systems, including equine primary post-natal brain-derived cells (EpBCs) and their immortalized counterparts (EiBCs). EpBCs were isolated from the hippocampus and successfully expanded, while immortalization via Simian virus 40 large T antigen generated EiBCs lines with distinct transcriptomic reprogramming characterized by cell cycle activation and partial dedifferentiation. Despite testing three non-integrating approaches—Sendai virus, episomal vectors, and synthetic mRNA—no induced pluripotent stem cells (iPSCs)-like colonies were detected under the tested conditions, possibly reflecting a combination of species-specific, cell-intrinsic, and donor-specific factors, as well as culture conditions, factor compatibility, and delivery efficiency. Together, these findings establish a foundational equine brain-derived cell platform and highlight key challenges in equine reprogramming. Its *One Health* relevance is prospective, as this system may support future comparative neurological research and the development of equine brain organoids.

## 1 Introduction

In contemporary veterinary medicine, attention to equine health has expanded substantially, particularly in the areas of infectious disease prevention and treatment (1–9). Among infectious diseases targeting the equine nervous system (ENS), the major zoonotic agents include Eastern, Western and Venezuelan equine encephalitis, as well as West Nile and Borna disease viruses (10–17). Additionally, equines develop several neurodegenerative disorders—such as Equine Degenerative Myeloencephalopathy and Neuroaxonal Dystrophy, Equine Motor Neuron Disease, Cerebellar Abiotrophy and Alzheimer-like neuropathology (1–9). These conditions closely parallel human disorders, underscoring the need for equine *in vitro* systems to investigate diseases of the ENS and support cross-species comparative research (18–21).

Two-dimensional (2D) cell culture systems and three-dimensional (3D) organoid models provide complementary platforms for such investigations (18–30). 2D cultures are typically established from primary somatic cells (pSCs) and because of their limited proliferative capacity, immortalization is required to support expansion (31–36). Immortalization strategies employ viral [e.g. Simian virus 40 large T antigen (SV40)] or non-viral (e.g., KRAS) oncogenes, often in combination with human telomerase reverse transcriptase (hTERT) (31–45). Despite their utility, 2D cultures lack the complexity of native nervous tissue (19–21, 29, 30, 46–53). Differently, organoids better recapitulate key aspects of organ-specific architecture and physiology (19, 20, 46–52). They may be generated from embryonic stem cells (ESCs), adult multipotent stem cells (aMSCs) or induced pluripotent stem cells (iPSCs) (18–20, 46–52, 54–61). Reprogramming to iPSCs relies on ectopic expression of Yamanaka’s factors (i.e., OCT4, SOX2, c-MYC and KLF4) by pSCs, delivered through integrating (retroviral or lentiviral) or non-integrating systems such as Sendai virus, adenoviral vectors, episomal plasmids, transposons or synthetic mRNAs (18, 54, 57, 58, 60–70).

Compared with human systems, veterinary NS *in vitro* resources remain limited. While human primary and immortalized NS cells, ESCs/iPSCs, and differentiation protocols are well established, animal NS models are mostly restricted to selected primary or immortalized cells generated in-house (45, 71–82). Additionally, although animal ESCs-like cultures and veterinary iPSCs have been reported, no commercial animal ESCs/iPSCs lines are available, iPSCs have not been derived from NS cells, and animal iPSCs differentiation and organoid technologies remain largely unexplored (35, 47, 83–89).

In light of the considerations stated above, this study aimed to generate equine immortalized post-natal brain-derived cells (EiBCs) through the immortalization of equine primary post-natal brain-derived cells (EpBCs). With the term “brain-derived,” we refer exclusively to the anatomical origin of the isolated cells, which were obtained from hippocampal tissue, and does not imply a defined neural or glial identity Furthermore, we attempted to reprogram EpBCs to equine induced pluripotent stem cells (EiPSCs) using Sendai virus, episomal plasmids and synthetic mRNAs (29, 90). The establishment of post-natal equine brain-derived *in vitro* models may provide a basis for future mechanistic studies of equine neuropathogens, comparative investigations of human neurological disorders, and translational applications within a *One Health* framework (18, 20, 29, 30).

## 2 Materials and methods

### 2.1 Isolation and establishment of equine primary post-natal brain-derived cells

A two-day-old female foal was euthanized intraoperatively due to intestinal agenesia at the University Veterinary Teaching Hospital of the University of Padua (Legnaro, Italy). Necropsy was performed immediately post-mortem at the adjacent Department of Comparative Biomedicine and Food Science of the University of Padua (Legnaro, Italy). Hippocampal tissue fragments (approximately 0.5 × 0.5 cm) were suspended in sterile phosphate-buffered saline (PBS) supplemented with 10% penicillin–streptomycin (P/S, Thermo Fisher Scientific, #15140122). Under aseptic conditions, brain biopsies were washed with PBS, dissected to remove visible blood vessels, and minced with a sterile razor blade. Tissue dissociation was performed using the Papain Dissociation System (Worthington Biochemical Corporation, #LK003150), following the manufacturer’s protocol. Briefly, samples were digested at 37 °C for 1.5 h, filtered through a 70 μm cell strainer and centrifuged at 300×g for 5 min. The resulting pellet was subjected to density gradient centrifugation with an albumin-ovomucoid inhibitor solution at 100×g for 5 min and plated in one well of a 24-well standard (sP1) or low adhesion plate (lP1) and cultured in Dulbecco’s Modified Eagle Medium/Nutrient Mixture F-12 (DMEM/F-12, Thermo Fisher Scientific, #11320033) with 1% GlutaMAX™ supplement (Glutamax, Thermo Fisher Scientific, #35050061), 1% MEM Non-Essential Amino Acids solution (NEAA, Thermo Fisher Scientific, #11140050), 1% N-2 supplement (N-2, Thermo Fisher Scientific, #17502048), 2% B-27™ supplement (B-27, Thermo Fisher Scientific, #17504044), 1% P/S, 20 ng/mL human basic fibroblast growth factor (hFGF, Thermo Fisher Scientific, #100-18B-50UG) and 20 ng/mL human epidermal growth factor (Merck, #E9644) (eBCsm1) in an atmosphere of humidified air and 5% CO_2_ at 37 °C. Four days after, the supernatant from the wells of sP1 and lP1 was centrifuged at 400 × *g* for 5 min and re-plated in two wells each of a 24-well standard (sP2) or low adhesion plate (lP2), respectively. Half of the wells of sP2 and lP2 were then cultured using eBCsm1 or eBCsm1 supplemented with 2% fetal bovine serum (FBS, Thermo Fisher Scientific, #A5256701) (eBCsm2). After 1 week, the supernatant from the wells of sP2 and lP2 was transferred to one well each of two 24-well standard plates (sP3a and sP3b, respectively). From this moment on, all wells were cultured in eBCsm2. After 5 days, the supernatant from the well of sP3a was centrifuged and re-plated in one well of a 24-well standard plate (sP4a). After 2 days, when EpBCs reached nearly 100% confluence in sP1 and sP2, cells were cultured for 5 days in eBCsm1. Then, supernatant from the wells of sP2 was transferred to one well of a 24-well low adhesion plate (lP3) and fresh eBCsm2 was replaced again in all wells. From the day after on, EpBCs were cultured in eBCsm1 supplemented with 10% FBS (eBCsm3). After 4 days from the addition of eBCsm3, when EpBCs reached nearly 100% confluence in sP1, cells were single washed with PBS and enzymatically detached using Accutase (Merck, #A6964) diluted (1:3) in PBS at 37 °C for 5 min. Then, EpBCs were centrifuged at 400 × *g* for 5 min and seeded (1:2) into one well each of a 24-well standard (sP5) or low adhesion plate (lP4) in eBCsm3 and eBCsm1, respectively. When cells reached nearly 100% confluence in sP5, EpBCs were split (1:2) and further expanded with a seeding rate ranging from 1:2 to 1:4. EpBCs were screened every 3 months for mycoplasma contamination using the MycoAlert® Mycoplasma Detection kit (Lonza, #LT07-703) and the Venor®GeM Classic (Minerva Biolabs, #11-1025) kit. A schematic overview of the methodology is available in the Supplementary Material (Figure S1).

### 2.2 Immortalization of equine primary post-natal brain-derived cells

For immortalization, EpBCs (passage 5) at 80% confluence were transfected using either GenJet™ *In Vitro* DNA transfection reagent (GenJet, SignaGen® Laboratories, #SL100488) or Lipofectamine™ 3000 transfection reagent (Lipofectamine 3000, Thermofisher Scientific, #L3000001) with plasmid DNA encoding SV40 (pSV3-neo, ATCC, #37150) or hTERT (pCl neo-hEST2, Addgene, #1781), following the manufacturer’s protocol. GenJet was removed 5 h post-transfection, whereas Lipofectamine 3000 was removed after 6 h (half-volume media replacement) or after 24 h. One 6-well each of EpBCs transfected with either a GFP-expressing plasmid (Monster Green® Fluorescent Protein Vector, Promega, #E6421) or incubated with transfection reagent only was included to evaluate transfection efficiency and cytotoxicity, respectively. One week post-transfection, EpBCs were subjected to selection with 350 μg/mL G418 antibiotic (Merck, #G8168-10ML) for 4 weeks.

Finally, EiBCs proliferation was evaluated at passage 10, 15, and 20 using the PrestoBlue™ Cell Viability Reagent (Themo Fisher Scientific, #A13261), following the manufacturer’s protocol. EiBCs were seeded at 3000 cells/well and analyzed at 0, 24, 48, and 72 h post-seeding (three technical replicates) after a 15 min incubation with the reagent. Background-corrected values were used to generate growth curves and population doubling time (PDT) was estimated from the slope (k) of the linear regression of ln-transformed values versus time, according to PDT = ln (2)/*k*.

### 2.3 Protein extraction, Western Blot, immunofluorescence and karyotyping analyses of equine primary and immortalized brain-derived cells

To characterize EpBCs (passage 5) and EiBCs (passage 10), qualitative validation of antibodies was performed by Western Blot (WB). Orthologous sequences were identified as the reciprocal best hits in GenBank through BLASTp searches (91). Sequences with *E*-value = 0.0 and 100% coverage were aligned with CLUSTALW to assess conservation level (92). Proteins showing ≥65% sequence identity were therefore selected WB (Supplementary Table S1).

For protein extraction, cells at 100% confluence were lysed for 5 min using 120 μL of Pierce™ RIPA buffer (Thermo Fisher Scientific, #89901) supplemented with cOmplete Mini, EDTA-free protease inhibitor cocktail tablets (Roche, #11836170001), following the manufacturer’s protocol. Cell lysates were then centrifuged at 4 °C at 14000×g for 15 min. Protein concentrations were calculated using the Pierce™ BCA protein Assay kit (Thermo Fisher Scientific, #23225), following the manufacturer’s protocol.

For WB, 15 μg of proteins were first denatured at 70 °C for 10 min or at 95 °C for 5 min and then resolved using Bolt™ Bis-Tris Plus Mini protein gels, 4–12% (Thermo Fisher Scientific, #NW04125BOX) or NuPAGE™ Tris-Acetate Mini protein gels, 3–8% (Thermo Fisher Scientific, #EA03755BOX) and transferred to a nitrocellulose membrane using the iBlot™ Transfer Stack kit (Thermo Fisher Scientific, #IB301002). To verify equal protein loading and transfer efficiency, membranes were stained with AdvanStain Ponceau (Aurogene, #R-03021-D50) for 5 min at room temperature. To block non-specific binding sites, blots were incubated for 90 min in 5–10% non-fat dry milk in Tris-buffered saline (TBS) containing 0.05–2% Tween-20 (TBS-T) at room temperature. Then, blots were incubated at 4 °C overnight with selected primary antibodies diluted in TBS-T containing 1–5% non-fat dry milk (Supplementary Table S1). Then, membranes were incubated with goat anti-rabbit IgG (H + L) poly-HRP secondary antibody (1:3000, Thermo Fisher Scientific, #32260) or goat anti-mouse IgG (H + L) poly-HRP secondary antibody (1:3000, Thermo Fisher Scientific, #32230) diluted in TBS-T for 1 h at room temperature. Reactive bands were visualized using the SuperSignal™ West Pico PLUS Chemiluminescent Substrate kit (Thermo Fisher Scientific, #34577). Proteins extracted from canine tissues (brain, spleen and smooth muscle), canine mammary tumor and *Tursiops truncatus* immortalized fibroblast cells were included as positive controls.

For immunofluorescence (IF), EpBCs (passage 5) and EiBCs (passage 10) at 60% confluence were fixed with 4% paraformaldehyde solution for 20 min at room temperature. Cells were then permeabilized with PBS containing 0.1% Triton X-100 for 15 min at room temperature. To block non-specific binding sites, cells were incubated with PBS containing 4% bovine serum albumin for 1 h at room temperature and then at 4 °C overnight with selected primary antibodies (Supplementary Table S1). Cells were then incubated with goat anti-mouse IgG (H + L) Alexa Fluor® 647 (1:200–1:1000, Aurogene, #AB150115-500UG) for 1 h at room temperature in the dark. Nuclei were counterstained with Hoechst33342 for 10 min (Thermo Fisher Scientific, #62249) and imaged using THUNDER Imager 3D Assay (Leica).

To assess genomic integrity, EiBCs (passage 10) at 50% confluence, were treated with 0.1 μg/mL colcemide (Biochrom AG, #L6221) for 2.5 h. Then EiBCs were detatched with Trypsin–EDTA (0.05%/0.02%) (Biochrom AG, #L2143), centrifuged at 300×g for 10 min and incubated in potassium chloride for 20 min at 37 °C, fixed and washed with an acetic acid/96% methanol solution (3:1). Samples were then sent to University of Jena (Jena, Germany) for analyses. Results were then compared to the already available dataset present about equine species karyotype (93).

### 2.4 RNA extraction, quantification and analysis of equine primary and immortalized brain-derived cells

For RNA extraction, EpBCs (passage 5, 7 and 9) and EiBCs (passage 10, 12 and 14) at 100% confluence were detached and centrifuged. The obtained cell pellet was snap frozen at −80 °C until acid nucleic extraction was performed using the miRNAeasy Micro kit (Qiagen, #217084), following the manufacturer’s protocol. Extracted RNA was then quantified using both Nanodrop ND-1000 technology and the Qbit™ RNA BR Assay kit (Thermo Fisher Scientific, #Q10211). Samples were sent for RNA-sequencing (RNA-seq) to Biomarker Technologies (BMK) GmbH (Münster, Germany).

For RNA-seq analysis, a total of six RNA-seq libraries derived from equine post-natal brain-derived cells were processed using the nf-core/rnaseq pipeline v3.22.0^1^. Sequencing reads were aligned to the *Equus caballus* reference genome (NCBI assembly GCA_041296265.1) using STAR. Gene-level quantification was performed with RSEM, generating raw read counts for each gene. The resulting gene-level raw count matrix was analyzed using iDEP (v2.4.4). Lowly expressed genes were filtered out by retaining those with counts ≥0.5 counts per million in at least one sample. The internal routine of DESeq2 “estimateSizeFactor” was then used to estimate the scaling factors for each of the samples. After estimating the scaling factors, the raw counts were subjected to differential expression analysis. Gene identifiers were mapped to Ensembl/STRING databases and differential expression analysis was conducted using DESeq2 with the standard Wald test and independent filtering enabled. Genes were considered differentially expressed if they met a false discovery rate (FDR) threshold of 0.05 and a minimum fold-change (FC) of 2. Functional enrichment analysis was performed using Gene Set Enrichment Analysis (GSEA, pre-ranked mode) across Gene Ontology Biological Process (GOBP), Molecular Signatures Database Hallmark (MSigDB Hallmark) and Kyoto Encyclopedia of Genes and Genomes (KEGG) set collections, with pathway sizes restricted to 5–2000 genes and an FDR cutoff of 0.1. Directionality of FC was preserved. Similarly, to identify significantly enriched cellular signatures, a Signature custom made database was realized using gene expression data obtained from the DropViz resource^2^, based on the single-cell transcriptomic atlas of the adult mouse brain (94) (Supplementary Table S2). To assess the presence of plasmid-derived transcripts in the RNA-seq datasets, paired-end reads from each replicate were independently aligned against the complete plasmid reference sequence using Bowtie 2 v2.5.5 (95), executed within a custom Apptainer container. The plasmid reference was indexed using bowtie2-build, and read alignment was performed in local mode using the—very-sensitive-local preset, 16 computational threads, and the—no-unal option. The resulting alignments were processed using SAMtools v1.24 (96). Reads were filtered to retain mapped alignments with a mapping quality of at least 20 (-q 20 -F 4), converted to BAM format, coordinate-sorted, and indexed. The number of retained alignments, number of covered bases, percentage of the plasmid sequence covered, mean sequencing depth, and mean mapping quality were calculated using samtools view and samtools coverage. Per-base sequencing depth, including positions with zero coverage, was obtained using samtools depth -aa. Samples were processed separately, and mapping profiles were compared between experimental conditions. Evidence of plasmid-derived transcription was evaluated by considering the number of aligned reads together with their distribution across the plasmid, coverage breadth, sequencing depth, mapping quality, and consistency among replicates.

### 2.5 Reprogramming of equine primary post-natal brain-derived cells to equine induced pluripotent stem cells

To reprogram EpBCs to EiPSCs, three non-integrating reprogramming systems were chosen, being Sendai virus, episomal plasmids and synthetic mRNA.

For Sendai virus reprogramming, EpBCs (passage 5) at 50% confluence, were transduced with the CytoTune™-iPS 2.0 Sendai Reprogramming kit (Thermo Fisher Scientific, #A16517) encoding OCT4, SOX2, c-MYC and KLF4, following manufacturer’s protocol. Multiplicities of infection (MOIs) of 5-5-3/6, 10-10-5/10, 15-15-5/10 and 20-20-5/10 were employed to transduce EpBCs.

For episomal plasmids reprogramming, EpBCs (passage 5) at 100% confluence were detached and centrifuged. Cells were then resuspended in 80 μL of Opti-MEM™ (Thermo Fisher Scientific, #31985062) at a cell density of 5–10 × 10^6^ cells/mL. EpBCs were then transfected with 8 μg each of plasmid DNA encoding a GFP-expressing plasmid (Monster Green® Fluorescent Protein Vector, Promega, #E6421), OCT4 (pCXLE-hOCT3/4-shp53-F, Addgene, #27077), NANOG, LIN28 and c-MYC (pCXLE-hMLN, Addgene, #27079) and SOX2 and KLF4 (pCXLE-hSK, Addgene, #27078) using the ECM®630 Electroporation System (BTX, Harvard Apparatus), applying the following electroporation parameters: 300 V, 50 μF, 1,575 Ω and 2 pulses.

After 1 week, EpBCs were detached and replated onto either CF1 mouse embryonic fibroblasts (MEFs, Thermo Fisher Scientific, #A34181) or Geltrex™ LDEV-free reduced growth factor basement membrane matrix (Geltrex, Thermo Fisher Scientific, #A1413201) coated plates. From the day after, EpBCs were maintained in two different pluripotency cell culture media (EiPSCsm1 or EiPSCsm2) with three different cytokine formulations (F1, F2 or F3) for 3 weeks (Supplementary Table S3).

For synthetic mRNA reprogramming, EpBCs (passage 5) at 70% confluence were transfected with either the StemRNA™ 3rd Gen Reprogramming kit (StemRNA kit, Reprocell, #00-0076) or the StemMACS iPSC mRNA Repro. kit, human (StemMACS kit, Miltenyi Biotec, #130-132990), following the manufacturer’s protocol. Adaptation to kit-supplemented or F2 cell culture media was tested and transfection intervals were extended to 36 h with doubled mRNA amounts. In addition, one transfection per day and 6 h reduced exposure were also applied to the StemRNA kit and the StemMACS kit, respectively. One 12-well of EpBCs incubated with transfection reagent was included to evaluate transfection cytotoxicity.

BJ human skin fibroblasts were included as positive controls for all reprogramming experiments. In all experiments, untransfected cells were included as negative controls to assess EpBCs health.

## 3 Results

### 3.1 eBCsm3 cell culture media enables the growth of equine primary post-natal brain-derived cells

The day following plating, undigested tissue fragments were observed in all wells of both sP1 and lP1 cultures. After 48 h, EpBCs began to adhere exclusively in sP1 wells. Eight days post-isolation, EpBCs were also detected in sP2 wells cultured in eBCsm2. Cells in sP1 wells appeared more heterogeneous, whereas those in sP2 had predominantly elongated fibroblast-like morphology (Supplementary Figure S2A). Eighteen days after tissue dissociation, when EpBCs in sP1 and sP2 had reached approximately 100% confluence, cells were switched to eBCsm1 for 5 days. Within 24 h, spheroids resembling neurospheres emerged across all wells. However, these aggregates exhibited signs of cellular detachment, prompting a media change back to eBCsm2. To promote recovery, EpBCs were subsequently maintained in eBCsm3. Four days after, EpBCs in sP1 cultures reached near-complete confluence and were expanded as previously described.

### 3.2 Simian virus 40 large T antigen mediates the immortalization of equine primary post-natal brain-derived cells

Following transfection, hTERT-treated EpBCs showed marked cytotoxicity, whereas pSV3-neo-transfected cells displayed better tolerance. Therefore, protocol optimization was continued using pSV3-neo with GenJet and Lipofectamine 3000. Reducing Lipofectamine 3000 exposure from 24 h to 6 h mitigated reagent-associated toxicity, and both optimized approaches achieved >80% GFP-positive cells. After G418 selection, resistant EiBCs colonies appeared within 10 days, showing a more homogeneous, rounded and compact morphology compared with EpBCs (Supplementary Figure S2B). One EiBCs line was expanded with a splitting ratio between 1:10 and 1:20 until passage 20 Interestingly, PrestoBlue signal increased over time at passage 10, 15, and 20, indicating maintained proliferative activity up to passage 20 (Supplementary Figure S3). Estimated PDTs were 15.37 h at passage 10, 18.21 h at passage 15, and 26.41 h at passage 20, corresponding to a mean PDT of 20.0 ± 5.7 h across the investigated passages. Although proliferation was slower at passage 20, EiBCs retained proliferative capacity throughout the investigated passage range.

### 3.3 Equine primary and immortalized post-natal brain-derived cells express glial and epithelial phenotypic markers at Western Blot and immunofluorescence

At WB, alpha-smooth muscle actin (SMA), a 42 kDa protein, was detected in protein extracts from both EpBCs (Figure 1A) and EiBCs (Figure 1B). The mesenchymal cell marker vimentin (VIM), a 54–57 kDa protein, was detected in protein extracts from both EpBCs (Figure 1C) and EiBCs (Figure 1D). Glial fibrillary acidic protein (GFAP) and pan-keratin (panCK), 52 and 40–56.5 kDa proteins marking glial and epithelial cells, were detected in EpBCs (Figure 1E) and EiBCs, respectively. (Figure 1F). Synaptophysin (Figures 1G,H), neurofilament 200 (Figures 1I,J) and Von Willebrand factor (Figures 1K,L), 40, 200 and 500–10000 kDa proteins, respectively were detected neither in EpBCs nor in EiBCs. Full gel images are available in Supplementary Figure S4.

**Figure 1:**
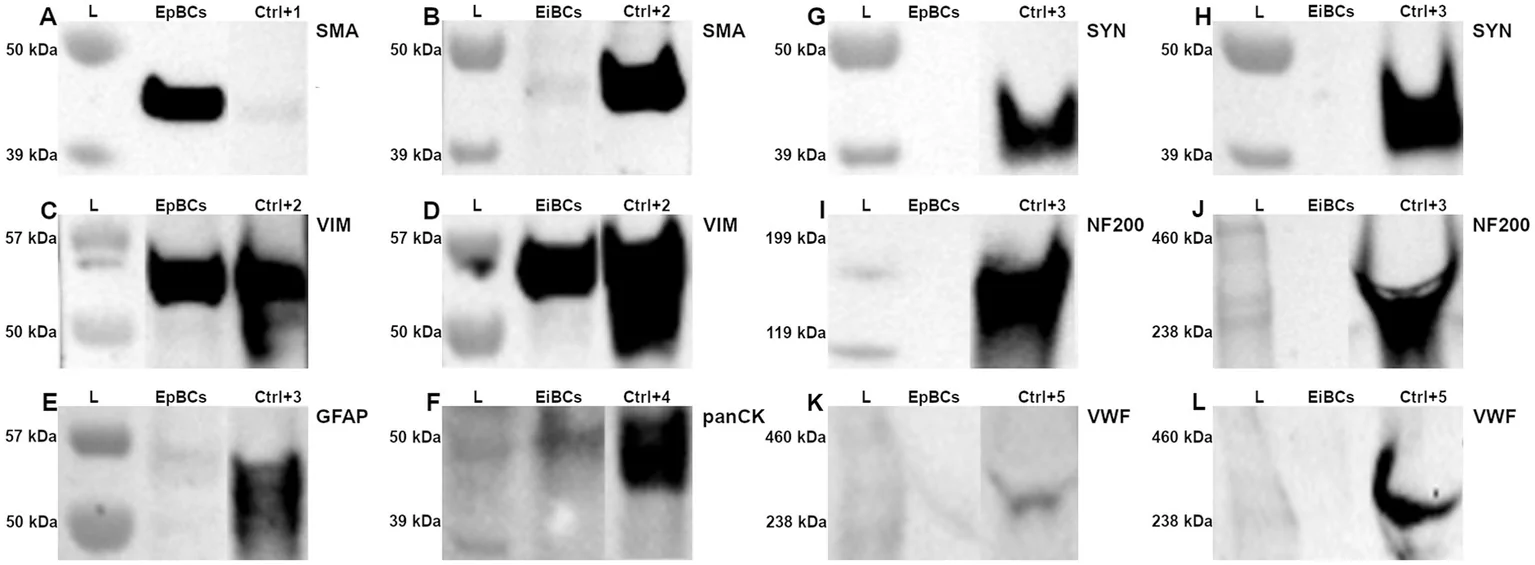
Western Blot expression of alpha-smooth muscle actin (SMA, **(A,B)**), vimentin (VIM, **(C,D)**), glial fibrillary acidic protein (GFAP, **(E)**), pan-keratin (panCK, **(F)**), synaptophysin (SYN, **(G,H)**), neurofilament 200 (NF200, **(I,J)**) and Von Willebrand factor (VWF, **(K,L)**) in equine primary and immortalized post-natal brain-derived cells, related to Supplementary Figure S3. L, ladder; EpBCs, equine primary brain-derived cells; Ctrl+1, canine smooth muscle; EiBCs, equine immortalized post-natal brain-derived cells; Ctrl+2, *Tursiops truncatus* immortalized fibroblast cells; Ctrl+3, canine brain; Ctrl+4, canine mammary tumor cells; Ctrl+5, canine spleen.

At IF, cytoplasmic SMA positivity was detected in approximately 20% of EpBCs and 10% of EiBCs (Figures 2A,B), while cytoplasmic VIM expression was detected in 100% of both EpBCs and EiBCs (Figures 2C,D). Cytoplasmic GFAP positivity was observed in approximately 50% of EpBCs (Figure 2E), whereas cytoplasmic panCK expression was detected in 100% of EiBCs (Figure 2F). These percentages represent descriptive estimates based on the analyzed representative fields.

**Figure 2:**
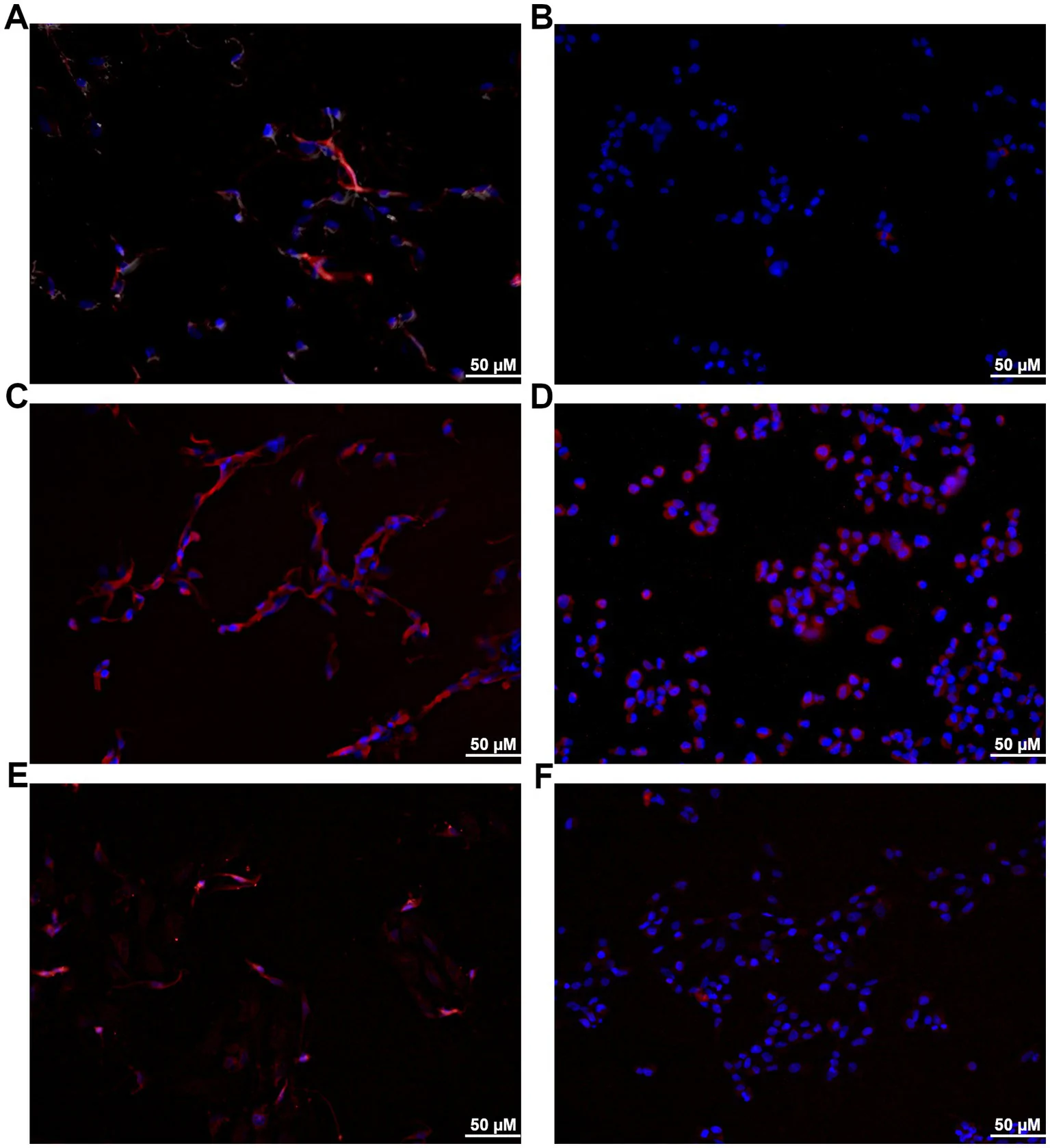
Immunofluorescence expression of alpha-smooth muscle actin **(A,B)**, vimentin **(C,D)**, glial fibrillary acidic protein **(E)** and pan-keratin **(F)** in equine primary and immortalized post-natal brain-derived cells (20×).

Following karyotype analysis, EiBCs exhibited a normal chromosomal complement consistent with that reported for *E. caballus* (93) (Supplementary Figure S5).

### 3.4 Equine immortalized post-natal brain-derived cells show activation of cell cycle mitotic pathways and a less heterogeneous cell composition compared to equine primary post-natal brain-derived cells

Sequencing generated on average 24 M high quality raw-reads, which were then mapped onto the horse genome with an average mapping rate of ~93%. Following preprocessing steps, 34,610 unique genes were detected across the samples, of which 16,251 passed the filtering criteria and were retained for downstream differential expression analysis. An initial unsupervised analysis was conducted by means of principal component analysis (PCA) which demonstrated that the primary source of the total variance (PC1, 91.83%) segregated EiBCs from EpBCs, whereas the secondary remaining (PC2, 2.7%) of the total variance was attributable to intra-group differences (Figure 3A), driven by different genes (Figures 3B,C). Differential expression analysis revealed marked transcriptional differences between EpBCs and EiBCs with 5,417 of differentially expressed genes (2,241 upregulated and 3,176 downregulated). Full list of up and downregulated genes is available in Supplementary Table S4. Among the differentially expressed genes with the highest FC (i.e., upregulated), genes such as *KRT3*, *KRT15*, *INSC*, *SLFN11*, *PLA2G3*, *GPRC5A*, *DDN*, *SCIN* and *CCNB3* were found, while genes such as *SMA*, *GFAP*, *KRT1*, *KRT18*, *ERBB3* and *DIRAS3* were found among the top down-regulated in EiBCs compared to EpBCs. To gain an insight of the different pathways between the two populations, GSEA across GOBP, MSigDB Hallmark and KEGG consistently showed enrichment of cell-cycle, DNA replication, chromatin remodeling, DNA repair and proliferative/metabolic programs in EiBCs, together with downregulation of mesenchymal migration, Schwann cell proliferation and selected metabolic/signaling pathways (Figures 4A–C). Brain cell-type signature analysis showed predominant enrichment of brain-associated signatures in EpBCs, whereas EiBCs displayed a more neural stem cell (NSC)-like profile (Figure 4D). Full list of the pathways resulting from the GSEA against GOBP, MSigDB Hallmark, KEGG and Signature custom made database are available in Supplementary Table S5.

**Figure 3:**
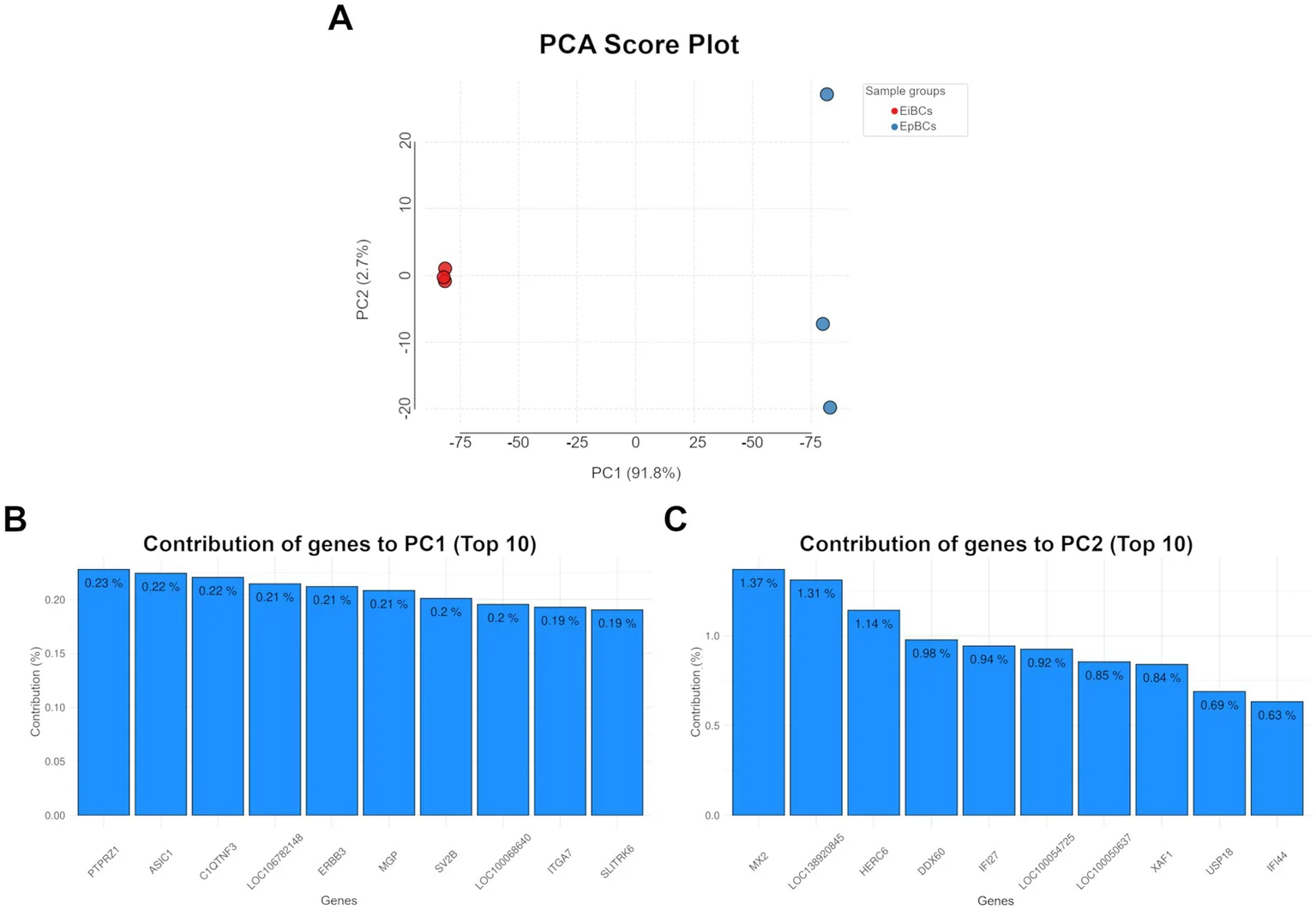
Principal component analysis showing the primary and secondary component of the total variance between equine primary (blue circles) and immortalized post-natal brain-derived cells (red circles) **(A)** together with the genes that were mostly responsible for the separation of groups and samples **(B,C)**. *x* axis: gene names; *y* axis: contribution (%).

**Figure 4:**
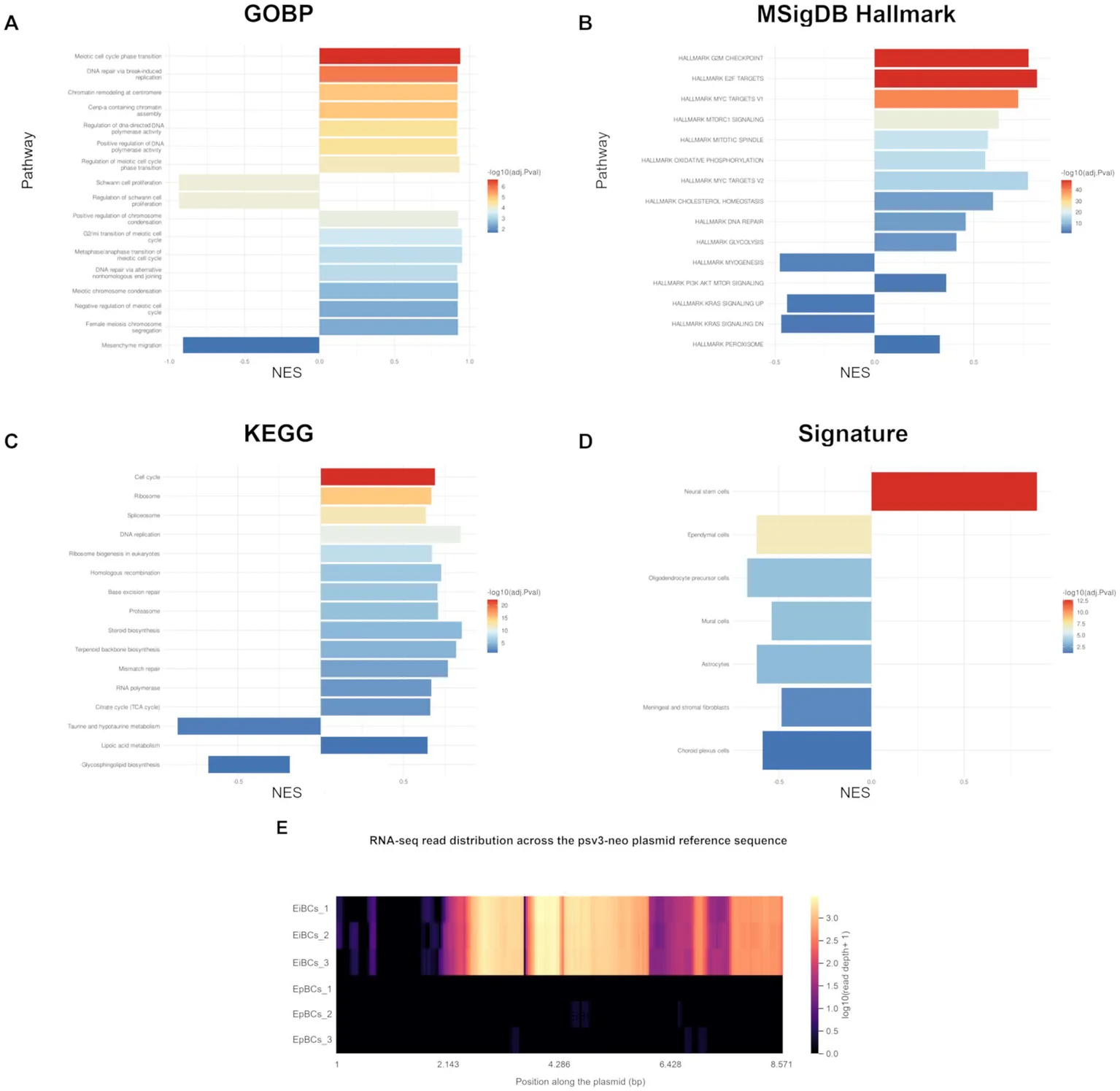
Histograms showing the gene set enrichment analysis resulting pathways run on the gene ontology biological process (GOBP, **(A)**), Molecular Signatures Database Hallmark (MSigDB Hallmark, **(B)**), Kyoto Encyclopedia of Genes and Genomes (KEGG, **(C)**) and Signature (Signature, **(D)**) custom made databases. *X* axis: Normalized Enrichment Score (NES); *y* axis: name of the pathway A representation of RNA-seq read distribution across the psv3-neo plasmid reference sequence across all samples is provided as well **(E)**. *X*-axis: position along the plasmid (bp); *y*-axis: equine primary and immortalized post-natal brain-derived cells (EiBCs_1, EiBCs_2 and EiBCs_3) and primary (EpBCs_1, EpBCs_2 and EpBCs_3) post-natal brain-derived cells.

Furthermore, alignment of RNA-seq reads against the complete pSV3-neo reference sequence revealed extensive plasmid-derived transcription in EiBCs (Figure 4E). Plasmid sequence coverage was highly consistent across the three passage-specific EiBCs samples, reaching 82.37, 83.54, and 83.20%, respectively. In contrast, EpBCs showed only negligible coverage, corresponding to 0, 4.15, and 5.12%. Per-base sequencing depth analysis confirmed broad and consistent coverage across large portions of the pSV3-neo sequence in EiBCs, whereas EpBCs displayed only sparse and isolated low-depth signals. Overall, these findings support the persistence of pSV3-neo-derived transcription in immortalized cells across the investigated passages. Importantly, RNA-seq coverage encompassed the SV40 large T antigen coding region, supporting persistent transcription of the immortalizing transgene in EiBCs. Raw count of RNA-sequencing reads of EiBCs and EpBCs is provided in Supplementary Table S6.

### 3.5 Sendai virus, episomal vectors and mRNA approaches do not enable the reprogramming of equine brain-derived cells to equine induced pluripotent stem cells

For all the three reprogramming methodologies, after 3 weeks from the transfection or transduction, no iPSCs-like morphology was detected. In particular, for all the MOIs of Sendai virus reprogramming, no morphological modifications were detected. In contrast, for episomal plasmid reprogramming, EpBCs showed a slight morphology change, homogeneously displaying a more elongated fibroblast-like shape particularly while being cultured with F2 on MEFs (Supplementary Figure S6). In none of the cases, negative controls showered signs of suffering. Transfection efficiency could only be evaluated for episomal methodologies, showing a 70% positivity for GFP of all cells (Supplementary Figure S7). In contrast, adverse cell morphology was detected 24 h after applying synthetic mRNAs for all the experimental conditions tested with both kits but more prominently with F2 using the StemMACS kit (Supplementary Figure S8), either in the transfected wells but also in the control ones. Total cell detachment was appreciated roughly 72 h after the beginning of the protocol.

## 4 Discussion

Recent global health crises have reinforced the interconnectedness of animal, human and environmental health, central to the *One Health* paradigm (18–20). In this study, we investigated the generation of equine EiBCs through the immortalization of EpBCs and explored their reprogramming to EiPSCs. These approaches may represent preliminary steps toward the future development of equine brain organoids (EBOs) as more complex and physiologically relevant brain models (18, 20, 29, 47, 48, 51, 54, 56, 61, 97). Although equine pSCs and immortalized somatic cells (iSCs) are available, brain cell models remain absent (Supplementary Tables S7S9). Notably, only three studies have reported the isolation of equine glial cells from the thoracic sympathetic chain ganglia, retina and embryonic brain tissue (98–100). In this study, the hippocampus was selected because it represents a postnatal neurogenic niche, being a potential reservoir of NSCs, whose extended lifespan and multipotent potential may render them more amenable to iPSCs reprogramming (75, 101–107). Brain dissociation strategies reported in human *in vitro* research include mechanical and enzymatic approaches, as well as commercial kits (105–107). Based on the literature, the best way to approach animal-derived NS cells was the application of mechanical dissociation and Papain digestion, which were therefore applied in this study (98–100, 105, 107–110). Furthermore, low adhesion plates were intended to enrich NSCs capable of forming neurospheres in eBCsm1 (75, 111). However, substantial cell death was observed under these culture conditions, suggesting that EpBCs did not retain progenitor-like characteristics (75, 111). Despite that, the heterogeneous morphology of EpBCs was consistent with previous reports following equine brain tissue digestion, as well as in human medicine and other veterinary species (98–100, 112–119).

For continuous cell establishment, immortalization via liposome transfection was selected due to its simplicity and cost-effectiveness, although its efficiency depends on the cellular endocytic capacity (35, 36, 120–122). EpBCs were transfected with either SV40 or hTERT, targeting the inhibition of the p53 pathway and maintenance of telomere length, respectively (34, 36–42, 44, 123–125). Previous studies have reported the immortalization of equine NS pSCs using SV40 delivered through retroviral transduction, as well as for other human and veterinary brain-derived cells (41, 71, 72, 75, 79, 81, 98, 109, 126–129). In our study, cells transfected with hTERT exhibited pronounced cytotoxicity and although co-expression of SV40 and hTERT is reported to enhance immortalization efficiency, hTERT-induced toxicity has been described in sensitive human cell types (34, 37). In addition, marked cytotoxicity was observed in EpBCs incubated with Lipofectamine 3000 under the manufacturer’s conditions. Therefore, reducing the incubation time with the reagent significantly mitigated this effect. EiBCs colonies exhibited a more homogeneous elongated and compact colony morphology, as also described in previous studies (71, 72, 75, 79, 81, 109, 126, 127). Interestingly, growth-curve analysis at three different passages confirmed that the cells retained proliferative capacity throughout the investigated passage range. The estimated PDTs (as well as the variability between them) were comparable to those reported for other immortalized cell lines and showed a progressive increase with passage number, suggesting a modest reduction in proliferation rate at later passages (130, 131). At WB and IF, EpBCs displayed SMA, VIM and GFAP expression, consistent with a glial-like phenotype, in agreement with markers previously used to characterize human and veterinary glial pSCs, including equine ones (98–100, 112, 132, 133). EiBCs showed SMA, VIM and panCK positivity, suggesting the acquisition of a mesenchymal–epithelial phenotype (133). These differences in protein expression are supported by the downregulation of genes such as *GFAP*, *SMA* and a shift in *KRT* gene expression patterns in EiBCs compared to EpBCs. Interstingly, despite the reported genomic unstability of immortalized lines, EiBCs displayed normal karyotype at the analyzed passage. Concurrently, EiBCs exhibited prominent upregulation of genes involved in cell cycle progression, DNA replication, and genomic maintenance. These findings, alongside *ERBB3* and *DIRAS3* downregulation and enrichment of an NSC-like signature in GSEA, support the transcriptional changes associated with oncogene-driven immortalization, also reported only in human studies (134, 135). Indeed, PCA revealed that immortalization was the dominant source of transcriptional variance, exceeding intra-group heterogeneity. The reduced heterogeneity observed in EiBCs may therefore also reflect selective pressure during transfection and antibiotic selection, favoring the expansion of a more homogeneous or clonally enriched subpopulation capable of surviving the procedure and maintaining the introduced construct. Furthermore, the broad and highly consistent coverage of the pSV3-neo sequence observed in EiBCs, together with the almost complete absence of plasmid-associated reads in EpBCs, provides molecular evidence of persistent transcription from the immortalization construct. The few reads detected in EpBCs were sparse and restricted to isolated plasmid regions, making genuine plasmid transcription unlikely and suggesting background or non-specific alignment. Conversely, the extensive and reproducible coverage observed in EiBCs is consistent with specific expression of plasmid-derived sequences. Although EpBCs and EiBCs provide accessible 2D platforms, their limited structural complexity, together with the biological alterations induced by SV40-mediated immortalization, may restrict their ability to fully recapitulate normal equine neural biology. In particular, SV40-mediated disruption of p53- and cell cycle-related pathways may promote genomic instability and contribute to the marked transcriptomic divergence observed between EiBCs and EpBCs, although the generated lines retained a normal karyotype in our study. Therefore, EiBCs should be interpreted with caution as models of normal equine neural biology, and their suitability may depend on the specific biological question being investigated. These limitations further support the future development of more physiologically relevant models, such as EBOs. However, EiPSCs and organoid technologies remain poorly developed, and EBOs have not yet been reported. Since most published EiPSCs have been generated from non-neural cells using integrating systems, we tested non-integrating Sendai virus-, episomal-, and synthetic mRNAs-based strategies in EpBCs to evaluate their feasibility as a genetically matched starting population. To the current literature, EiPSCs have been generated from fibroblasts, keratinocytes, chondrocytes and mesenchymal stem cells (MSCs), using integrating systems encoding human or mouse Yamanaka’s factors (90, 136–154). In this study, EpBCs were attempted to be reprogrammed to iPSCs because the use of cells originating from the same established pSCs ensures genetic uniformity, improving reproducibility (18–20, 46–52, 54–61). Differentiation protocols from both equine ESCs and EiPSCs have also been developed, but no differentiation protocols for the generation of equine brain-derived cells from EiPSCs have been described (143, 145, 148, 149, 151–153, 155–163). Only one study managed to obtain equine Schwann-like cells from MSCs (164). In this study, Sendai virus transduction, despite high MOIs and limited cytotoxicity, did not induce morphological reprogramming, suggesting limited tropism or insufficient transgene delivery in EpBCs (61, 165–169). Episomal plasmids induced only modest morphological changes, consistent with incomplete reprogramming, potentially due to the limited maintenance of the EBNA1/OriP-based pCXLE vectors in non-primate mammalian cells. In contrast, synthetic mRNAs-based approaches caused marked cytotoxicity also in reagent-only controls, indicating that the outcome of this approach could be attributed both to the toxicity related to the delivery protocol as well to biological factors (61, 165–169). Together, these findings indicate that a combination of species-specific, cell-intrinsic, and donor-specific factors, as well as culture conditions, factor compatibility, and delivery efficiency might have limited reprogramming under the tested conditions (61, 165–169).

In conclusion, we established the first post-natal equine brain-derived 2D *in vitro* models, including EpBCs and EiBCs, and systematically evaluated multiple strategies for the reprogramming of EpBCs to iPSCs. Although no iPSCs-like colonies were detected under the tested conditions, our findings identify several technical and biological barriers associated with generating pluripotent cells from ENS. These results provide a foundation for future efforts to develop equine pluripotent stem cell platforms and, ultimately, EBOs, with potential applications in translational neuroscience, veterinary medicine, and *One Health* research.

### 4.1 Limitations of the study

Future studies should further define EpBCs (e.g., trough investigation of neural progenitor-like cells markers) and EiBCs identity and heterogeneity using single-cell RNA-seq, and additional donor-derived lines. Moreover, although SV40 transduction resulted in G418-resistant colonies, the present preliminary study did not fully assess telomere-related changes, which would be of interest for future studies. Moreover, RNA-seq demonstrates transcript presence rather than protein expression or functional activity. Therefore, these findings should be interpreted as evidence of persistent SV40 transcription, rather than direct demonstration of SV40 large T antigen protein expression, which should be further assesses.

Additionally, a major limitation of the present study is the use of human reprogramming factors across all delivery platforms, which may have introduced species-specific barriers in equine cells. Human factors were selected because they are widely used in established equine reprogramming protocols and allowed direct comparison with previous studies. Furthermore, expression and functional activity of the delivered reprogramming factors, including OCT4, SOX2, KLF4, and c-MYC, were not directly assessed. Consequently, the present experiments cannot distinguish inefficient factor expression or activity from failure of the downstream reprogramming process itself. However, the unsuccessful reprogramming observed here should not be interpreted only as evidence of an inherent species-specific barrier, as donor age, genotype, epigenetic state, tissue condition, and culture history may also have contributed. Future work should therefore assess equine-specific or equine codon-optimized factors, together with optimized delivery systems, Sendai virus tropism and entrance in the cells, integration-based approaches, alternative starting cell types, and additional donors to better distinguish species-related effects from donor- and culture-dependent constraints (18, 54, 57, 58, 60, 62–70).

## Data Availability

The datasets presented in this study can be found in online repositories. The names of the repository/repositories and accession number(s) can be found in the article/Supplementary material.

## Footnotes

1
https://nf-co.re/rnaseq/3.23.0/

2
http://dropviz.org/
