# Supplementary material for: Equine (Equus caballus) primary post-natal brain-derived cells: immortalization and preliminary reprogramming attempts to induced pluripotent stem cells
Source: Front Vet Sci. 2026 Oct 7;13:1931938. doi: 10.3389/fvets.2026.1931938 (PMC13683745; doi:10.3389/fvets.2026.1931938)
Supplement: Supplementary file 1 [file Supplementary_file_1.docx]

**Supplementary Material - Document S1**

**Table S1.** Primary antibodies used in Western Blot (WB) and immunofluorescence (IF) to characterize equine primary brain-derived cells and equine immortalized brain-derived cells.

| **Antibody company, #catalogue (clone)** | **Equine**  **identity** | **Species** | **Antigen** | **MW**  **[kDa]** | **WB dilution** | **IF**  **dilution** | **Cross-reactivity** |
| --- | --- | --- | --- | --- | --- | --- | --- |
| **Dako,**  **#M7315**  **(DAK-SYNAP)** | 96.2 % | Mouse | Human synaptophysin | 40 | 1:500 | N/A | Human |
| **Dako,**  **#M3515**  **(AE1/AE3)** | 82.8 % | Mouse | Human pan-keratin | 40-56.5 | 1:800 | 1:25 | Human |
| **Dako,**  **#M0851**  **(1A4)** | 100% | Mouse | Human alpha-smooth muscle actin | <42 | 1:1000 | 1:100 | Human, bovine, chicken, and rodent |
| **Diagnostic BioSystems,**  **#MOB199-05**  **(6F2)** | 96% | Mouse | Human glial fibrillary acidic protein | 52 | 1:1000 | 1:50 | Human |
| **Dako,**  **#M0725**  **(V9)** | 98.1% | Mouse | Porcine vimentin | 54-57 | 1:5000 | 1:50 | Human, African green monkey, bovine, canine, equine, gerbil, hamster, rabbit, rat, and rhesus macaque |
| **Merck,**  **#N0142**  **(N52)** | 92.3% | Mouse | Human neurofilament 200 | 200 | 1:1000 | N/A | Human, hamster, monkey, mouse, pig, rabbit, and rat |
| **Dako,**  **#A0082 (polyclonal)** | 73.9% | Rabbit | Human Von Willebrand factor | 500-  10 000 | 1:1000 | N/A | Human chicken, cow, dog, horse, mouse, pig, and rat |

MW: molecular weight; N/A: not applicable

**Table S3.** Composition of pluripotency cell culture media EiPSCsm1, EiPSCsm2, F1, F2 and F3 used for reprogramming of equine primary brain-derived cells to equine induced pluripotent stem cells.

| **Medium** | **Composition** |
| --- | --- |
| **EiPSCsm1** | DMEM/F-12  20% KnockOut™ serum replacement (KO-SR, Thermo Fisher Scientific, #10828028)  1% GlutaMax  1% NEAA  1% P/S  2-mercaptoethanol (β-mercapto, 1:1000, Thermo Fisher Scientific, #31350010) |
| **EiPSCsm2** | DMEM/F-12/Neurobasal™ Medium (1:1) (Thermo Fisher Scientific, #21103049)  20% KO-SR  1% GlutaMax  1% NEAA  1% P/S  1% N-2  2% B-27  2-mercaptoethanol (β-mercapto, 1:1000, Thermo Fisher Scientific, #31350010) |
| **F1** | hESCsm or EiPSCsm2 additioned with:  20 ng/mL hFGF  20 ng/mL human leukemia inhibitory factor (hLIF, Thermo Fisher Scientific, #300-05-25UG)  1 mM valproic acid (VA, Reprocell, #04-0007).  100 ng/mL human stem cell factor (Thermo Fisher Scientific, #300-07-10UG)  20 nM forskolin (Reprocell, #04-0025) |
| **F2** | hESCsm or EiPSCsm2 additioned with:  20 ng/mL hFGF  20 ng/mL hLIF  1 mM VA  1 µM A83-01 (Reprocell, #04-0014)  1 µM PD0325901 (Reprocell, #04-0006)  3 µM CHIR99021 (Reprocell, #04-0004) |
| **F3** | hESCsm or EiPSCsm2 additioned with:  20 ng/mL hFGF  20 ng/mL hLIF  1mM VA |
