# Supplementary material for: Equine (Equus caballus) primary post-natal brain-derived cells: immortalization and preliminary reprogramming attempts to induced pluripotent stem cells
Source: Front Vet Sci. 2026 Oct 7;13:1931938. doi: 10.3389/fvets.2026.1931938 (PMC13683745; doi:10.3389/fvets.2026.1931938)
Supplement: Supplementary file 2 [file Supplementary_file_2.docx]

**Supplementary Material - Document S2**

**Table S7.** Commercially available and in-house isolated cancerous equine cells to the current literature.

|  | **Commercially available** | **In-house isolated** |
| --- | --- | --- |
| **Neoplasia** | Sarcoid | Carcinoma(1)^,^, lymphosarcoma(2), melanoma(2) and sarcoid(3–8) |

**Table S8.** Commercially available primary and immortalized non-cancerous equine cells.

| **Cell type** | **Site of origin of primary cells** | **Site of origin of immortalized cells (gene)** |
| --- | --- | --- |
| **Epithelial** |  |  |
| *Epithelial*  (no other-specified) | - Liver and lung | - Kidney (SV40 large T antigen, HPV E6/E7) and lung (HPV E6/E7) |
| **Mesenchymal** |  |  |
| *Endothelial* |  | - Lymphatic vessel (SV40 large T antigen) |
| *Fibroblast* | - Embryo and skin |  |
| *Mesenchymal stem cell* | - Adipose tissue | Skeletal muscle (SV40 large T antigen) |
| *Mesenchymal*  (no other-specified) | Peripheral blood |  |

SV40: Simian virus 40; HPV: human papillomavirus.

**Table S9.** In-house isolated primary and immortalized equine cells from current literature.

| **Cell type** | **Site of origin of primary cells** | **Site of origin of immortalized cells (gene)** |
| --- | --- | --- |
| **Epithelial** |  |  |
| *Epithelial*  (no other-specified) | - Abdominal fluid(9), bronchi(10–12), cornea(13,14), intestine(15), kidney(16–19), liver(20), lung(21), oviduct(22), retina(23), skin(24), sweat glands(25), trachea(26) and uterus(27,28) | - Kidney (SV40 large T antigen, HPV E6/E7)(16,17,19), lung (HPV E6/E7)(21) and oviduct (SV40 large T antigen)(22) |
| *Keratinocyte* | - Cornea(13,14), hoof(29) and skin(30) |  |
| **Mesenchymal** |  |  |
| *Endothelial* | - Aorta(31,32), cornea(14), intestine(33,34), lymphnode(35), terminal arch and pulmonary vessel(18,29,31,36,37) and umbilical artery, vein and ring(18,38,39) |  |
| *Fibroblast* | - Abdominal wall(18), bronchi(11), cornea(40), embryo(3), kidney(33) , lung(33), oral mucosa(41,42), periodontium(43), skin(4,8,33,38,41,44), synovial membrane(45), tendon(46) and uterus(27) | Skin (hTERT)(44) |
| *Mesenchymal stem cell* | - Adipose tissue(47–52), amnion(53,54), bone marrow(47,49,50,52,54–60), dental pulp(61), embryo(62), gingiva and periodontal ligament(43), hair follicles(63), hoof(64,65), ileum(66) , uterus(67), peripheral and umbilical cord blood(47,49,50,52,59,68,69), sternum(66) , synovial fluid and membrane(70), tendons(54,71), umbilical cord(52,54,72,73) and Wharton’s jelly(47,69) | Bone marrow (hTERT)(60) |
| *Mesenchymal*  (no other-specified) | Adipose tissue(74), bone(75,76), cartilage(77–80) gingiva and periodontal ligament(43), kidney(81), mammary gland(82,83), peripheral blood(84–89), periosteoum(90), placenta(18,91–93), skeletal and smooth muscle(94–97), synovial fluid and membrane(45,98), tendon(84,99,100) and pituitary gland(101) | Cartilage (SV40 large T antigen)(80), skeletal and smooth muscle (SV40 large T antigen)(95,96) and placenta(93) |

SV40: Simian virus 40 large T antigen; HPV: human papillomavirus; hTERT: human telomerase reverse transcriptase.

**References**

1.	Pardini LL, Aaronson LM, Hering PL, Pardini RS. Heterotransplantation of equine carcinoma cells in athymic (nude ) mice.

2.	Zachariasewycz E, Baliga V. Studies on cell lines derived from a horse with lymphosarcoma. *Cancer* (1974) 34:696–704. doi: 10.1002/1097-0142(197409)34:3<696::AID-CNCR2820340328>3.0.CO;2-F

3.	Finlay M, Yuan Z, Morgan IM, Campo MS, Nasir L. Equine sarcoids: Bovine Papillomavirus type 1 transformed fibroblasts are sensitive to cisplatin and UVB induced apoptosis and show aberrant expression of p53. *Vet Res* (2012) 43:81. doi: 10.1186/1297-9716-43-81

4.	Monod A, Koch C, Jindra C, Haspeslagh M, Howald D, Wenker C, Gerber V, Rottenberg S, Hahn K. CRISPR/Cas9-Mediated Targeting of BPV-1-Transformed Primary Equine Sarcoid Fibroblasts. *Viruses* (2023) 15: doi: 10.3390/v15091942

5.	Watson RE, England JJ, Larson KA. Cultural Characteristics of a Cell Line Derived from an Equine Sarcoid. (1972). 727–731 p. https://journals.asm.org/journal/am

6.	Watson RE, Larson KA. Detection of Tumor-Specific Antigens in an Equine Sarcoid Cell Line. (1974). 714–718 p.

7.	Yuan ZQ, Gault EA, Gobeil P, Nixon C, Campo MS, Nasir L. Establishment and characterization of equine fibroblast cell lines transformed in vivo and in vitro by BPV-1: Model systems for equine sarcoids. *Virology* (2008) 373:352–361. doi: 10.1016/j.virol.2007.11.037

8.	Hainisch EK, Jindra C, Reicher P, Miglinci L, Brodesser DM, Brandt S. Bovine Papillomavirus Type 1 or 2 Virion-Infected Primary Fibroblasts Constitute a Near-Natural Equine Sarcoid Model. *Viruses* (2022) 14: doi: 10.3390/v14122658

9.	James D. Lillich DMWR-MDMKSSPEGDDPBDSP. Intra-abdominal hyaluronan concentration in peritoneal fluid of horses with sudden signs of severe abdominal pain. *AJVR* (2011) 72:

10.	Legere RM, Allegro AR, Affram Y, da Silveira BP, Fridley JL, Wells KM, Oezguen N, Burghardt RC, Wright GA, Pollet J, et al. Equine bronchial epithelial cells are susceptible to cell entry with a SARS-CoV-2 pseudovirus but reveal low replication efficiency. *Am J Vet Res* (2023) 84:1–11. doi: 10.2460/ajvr.23.06.0132

11.	Abs V, Bonicelli J, Kacza J, Zizzadoro C, Abraham G. Equine bronchial fibroblasts enhance proliferation and differentiation of primary equine bronchial epithelial cells co-cultured under air-liquid interface. *PLoS One* (2019) 14: doi: 10.1371/journal.pone.0225025

12.	Abraham G, Zizzadoro C, Kacza J, Ellenberger C, Abs V, Franke J, Schoon HA, Seeger J, Tesfaigzi Y, Ungemach FR. Growth and differentiation of primary and passaged equine bronchial epithelial cells under conventional and air-liquid-interface culture conditions. *BMC Vet Res* (2011) 7:26. doi: 10.1186/1746-6148-7-26

13.	Haber M, Cao Z, Panjwani N, Bedenice D, Li WW, Provost PJ. Effects of growth factors (EGF, PDGF-BB and TGF-β1) on cultured equine epithelial cells and keratocytes: Implications for wound healing. *Vet Ophthalmol* (2003) 6:211–217. doi: 10.1046/j.1463-5224.2003.00296.x

14.	Mathes RL, Dietrich UM, Krunkosky TM, Hurley DJ, Reber AJ. Establishing a reproducible method for the culture of primary equine corneal cells. *Vet Ophthalmol* (2009) 12:41–49. doi: 10.1111/j.1463-5224.2009.00729.x

15.	Stewart AS, Schaaf CR, Veerasammy B, Freund JM, Gonzalez LM. Culture of equine intestinal epithelial stem cells after delayed tissue storage for future applications. *BMC Vet Res* (2022) 18: doi: 10.1186/s12917-022-03552-6

16.	Andoh K, Kai K, Matsumura T, Maeda K. Further Development of an Equine Cell Line that can be Propagated over 100 Times. (2009). 11–14 p.

17.	Maeda K, Yasumoto S, Tsuruda A, Andoh K, Kai K, Otoi T, Matsumura T. Establishment of a Novel Equine Cell Line for Isolation and Propagation of Equine Herpesviruses. (2007). 989–991 p.

18.	Watanabe S, Ishikawa Y, Hara H, Hanzawa K, Mukoyama H. A Method of Primary Cell Culture for Establishing Equine Long-Term Culture Cell Lines. (1997). 95–99 p.

19.	Oguma K, Ishida M, Maeda K, Sentsui H. Efficient propagation of equine viruses in a newly established equine cell line, FHK-Tcl3.1 cells. *Journal of Veterinary Medical Science* (2013) 75:1223–1225. doi: 10.1292/jvms.12-0450

20.	Bakala A, Karlik W, Wiechetek M. Preparation of equine isolated hepatocytes. *Toxicology in Vitro*. Elsevier Ltd (2003). p. 615–621 doi: 10.1016/S0887-2333(03)00112-7

21.	Thorsteinsdóttir L, Torsteinsdóttir S, Svansson V. Establishment and characterization of fetal equine kidney and lung cells with extended lifespan. Susceptibility to equine gammaherpesvirus infection and transfection efficiency. *In Vitro Cell Dev Biol Anim* (2016) 52:872–877. doi: 10.1007/s11626-016-0046-9

22.	Dobrinski I, Jacob JR, Tentrant BC, Ball3 BA. GENERATION OF AN EQUINE OVIDUCTAL EPITHELIAL CELL LINE FOR THE STUDY OF SPERM-OVIDUCT INTERACTIONS.

23.	Szober CM, Hauck SM, Euler KN, Fröhlich KJH, Alge-Priglinger C, Ueffing M, Deeg CA. Profound re-organization of cell surface proteome in equine retinal pigment epithelial cells in response to in vitro culturing. *Int J Mol Sci* (2012) 13:14053–14072. doi: 10.3390/ijms131114053

24.	Borena BM, Meyer E, Chiers K, Martens A, Demeyere K, Broeckx SY, Duchateau L, Spaas JH. Sphere-forming capacity as an enrichment strategy for epithelial-like stem cells from equine skin. *Cellular Physiology and Biochemistry* (2014) 34:1291–1303. doi: 10.1159/000366338

25.	Wilson SM PJKWBDKSMIBUSGEHJDM. Investigation of stimulus-secretion coupling in equine sweat gland epithelia using cell culture techniques.  . *J Exp Biol 1993 Oct;183:279-99*

26.	Shibeshi W, Abraham G, Kneuer C, Ellenberger C, Seeger J, Schoon HA, Ungemach FR. Isolation and culture of primary equine tracheal epithelial cells. *In Vitro Cell Dev Biol Anim* (2008) 44:179–184. doi: 10.1007/s11626-008-9099-8

27.	Watson ED, Aubrey ES, Zanecosky HG, Sertich PL. Isolation and culture of glandular epithelial and stromal cells from the endometrium of mares. https://academic.oup.com/reproduction/article/95/1/269/8278377

28.	Barry A. Ball DPSPBDMPGATBvsPGMMJEEDP. Development to blastocysts of one- to two-cell equine embryos after coculture with uterine tubal epithelial cells. *Am J Vet Res* (1993) 54:

29.	Denise Wunn DMKJWDMKMPJKDPCRDM. Culture and characterization of equine terminal arch endothelial cells and hoof keratinocytes. *AJVR* (1999) 60:

30.	Alkhilaiwi F, Wang L, Zhou D, Raudsepp T, Ghosh S, Paul S, Palechor-Ceron N, Brandt S, Luff J, Liu X, et al. Long-term expansion of primary equine keratinocytes that maintain the ability to differentiate into stratified epidermis. *Stem Cell Res Ther* (2018) 9: doi: 10.1186/s13287-018-0918-x

31.	John T, Turek J, Lamar CH, Fessler JF, Bottoms GD. para para Cons Cons Ultrastructure of equine endothelial cells exposed 97 97.

32.	Finding EJT, Faulkner A, Nash L, Wheeler-Jones CPD. Equine Endothelial Cells Show Pro-Angiogenic Behaviours in Response to Fibroblast Growth Factor 2 but Not Vascular Endothelial Growth Factor A. *Int J Mol Sci* (2024) 25: doi: 10.3390/ijms25116017

33.	Ólafsdóttir G, Svansson V, Ingvarsson S, Marti E, Torsteinsdóttir S. In vitro analysis of expression vectors for DNA vaccination of horses: The effect of a Kozak sequence. *Acta Vet Scand* (2008) 50: doi: 10.1186/1751-0147-50-44

34.	Campbell-Thompson M. Secretagogue-induced [14C]aminopyrine uptake Secretagogue-induced [14C]aminopyrine uptake in isolated equine parietal cells in isolated equine parietal cells.

35.	Lugo T, Myers S, Nguyen TA. Isolation and Characterization of Equine Lymph Node Endothelial Cells. *Vet Sci* (2025) 12: doi: 10.3390/vetsci12090905

36.	MacEACHERN KE. Methods for the isolation, culture and characterisation of equine pulmonary artery endothelial cells. (1997). 147 p.

37.	John HA, La¥ing AJ, Marrs J, Baird A, Jat PS, Holdstock NB, Rossdale PD. Establishment and characterization of equine autonomic ganglion cell lines to enable direct testing of candidate toxins involved in equine dysautonomia (grass sickness).

38.	Brindley MA, Zhang B, Montelaro RC, Maury W. An Equine Infectious Anemia Virus Variant Superinfects Cells through Novel Receptor Interactions. *J Virol* (2008) 82:9425–9432. doi: 10.1128/jvi.01142-08

39.	Lessiak U, Melchert M, Walter I, Kummer S, Nell B, Tschulenk W, Pratscher B. Isolation-protocol, characterization, and in-vitro performance of equine umbilical vein endothelial cells. *Front Vet Sci* (2024) 11: doi: 10.3389/fvets.2024.1421946

40.	Marlo TL, Giuliano EA, Tripathi R, Sharma A, Mohan RR. Altering equine corneal fibroblast differentiation through Smad gene transfer. *Vet Ophthalmol* (2018) 21:132–139. doi: 10.1111/vop.12485

41.	Watts EJ RMT. Extracellular matrix expression by equine oral and limb fibroblasts in in vitro culture. . *Res Vet Sci 2012 Apr;92(2):213-8*

42.	Watts AE, Yeager AE, Kopyov O V., Nixon AJ. Fetal derived embryonic-like stem cells improve healing in a large animal flexor tendonitis model. *Stem Cell Res Ther* (2011) 2: doi: 10.1186/scrt45

43.	Mensing N, Gasse H, Hambruch N, Haeger JD, Pfarrer C, Staszyk C. Isolation and characterization of multipotent mesenchymal stromal cells from the gingiva and the periodontal ligament of the horse. *BMC Vet Res* (2011) 7: doi: 10.1186/1746-6148-7-42

44.	Vidale P, Magnani E, Nergadze SG, Santagostino M, Cristofari G, Smirnova A, Mondello C, Giulotto E. The catalytic and the RNA subunits of human telomerase are required to immortalize equid primary fibroblasts. *Chromosoma* (2012) 121:475–488. doi: 10.1007/s00412-012-0379-4

45.	Allison H. Kilborne dvm Hayam Hussein bvetmed mvsc, phd ALB dvm, phd. Effects of hyaluronan alone or in combination with chondroitin sulfate and N-acetyl-d-glucosamine on lipopolysaccharide challenge-exposed equine fibroblast-like synovial cells. *AJVR* (2017) 78:

46.	Rich T, Henderson LB, Becker DL, Cornell H, Patterson-Kane JC. Indicators of replicative damage in equine tendon fibroblast monolayers. *BMC Vet Res* (2013) 9: doi: 10.1186/1746-6148-9-180

47.	Merlo B, Teti G, Lanci A, Burk J, Mazzotti E, Falconi M, Iacono E. Comparison between adult and foetal adnexa derived equine post-natal mesenchymal stem cells. *BMC Vet Res* (2019) 15: doi: 10.1186/s12917-019-2023-5

48.	Tidd N, Michelsen J, Hilbert B, Quinn JC. Minicircle mediated gene delivery to canine and equine mesenchymal stem cells. *Int J Mol Sci* (2017) 18: doi: 10.3390/ijms18040819

49.	Pennington MR, Curtis TM, Divers TJ, Wagner B, Ness SL, Tennant BC, Van De Walle GR. Equine Mesenchymal Stromal Cells from Different Sources Efficiently Differentiate into Hepatocyte-Like Cells. *Tissue Eng Part C Methods* (2016) 22:596–607. doi: 10.1089/ten.tec.2015.0403

50.	Harman RM, Patel RS, Fan JC, Park JE, Rosenberg BR, Van de Walle GR. Single-cell RNA sequencing of equine mesenchymal stromal cells from primary donor-matched tissue sources reveals functional heterogeneity in immune modulation and cell motility. *Stem Cell Res Ther* (2020) 11: doi: 10.1186/s13287-020-02043-5

51.	Marycz K, Bourebaba N, Serwotka-Suszczak A, Mularczyk M, Galuppo L, Bourebaba L. In Vitro Generated Equine Hepatic-Like Progenitor Cells as a Novel Potent Cell Pool for Equine Metabolic Syndrome (EMS) Treatment. *Stem Cell Rev Rep* (2023) 19:1124–1134. doi: 10.1007/s12015-023-10507-3

52.	Chrisoula A. Toupadakis BAWPDCGPWKCBDLBDPGLFDLDGDJKLPSDODCEYP. Comparison of the osteogenic potential of equine mesenchymal stem cells from bone marrow, adipose tissue, umbilical cord blood, and umbilical cord tissue. *AJVR* (2010) 71:

53.	Violini S, Gorni C, Pisani LF, Ramelli P, Caniatti M, Mariani P. Isolation and differentiation potential of an equine amnion-derived stromal cell line. *Cytotechnology* (2012) 64:1–7. doi: 10.1007/s10616-011-9398-x

54.	Lovati AB, Corradetti B, Lange Consiglio A, Recordati C, Bonacina E, Bizzaro D, Cremonesi F. Comparison of equine bone marrow-, umbilical cord matrix and amniotic fluid-derived progenitor cells. *Vet Res Commun* (2011) 35:103–121. doi: 10.1007/s11259-010-9457-3

55.	Guest DJ, Smith MRW, Allen WR. Equine embryonic stem-like cells and mesenchymal stromal cells have different survival rates and migration patterns following their injection into damaged superficial digital flexor tendon. *Equine Vet J* (2010) 42:636–642. doi: 10.1111/j.2042-3306.2010.00112.x

56.	Jammes M, Cassé F, Velot E, Bianchi A, Audigié F, Contentin R, Galéra P. Pro-Inflammatory Cytokine Priming and Purification Method Modulate the Impact of Exosomes Derived from Equine Bone Marrow Mesenchymal Stromal Cells on Equine Articular Chondrocytes. *Int J Mol Sci* (2023) 24: doi: 10.3390/ijms241814169

57.	Mitchell A, Rivas KA, Smith R, Watts AE. Cryopreservation of equine mesenchymal stem cells in 95 % autologous serum and 5 % DMSO does not alter post-thaw growth or morphology in vitro compared to fetal bovine serum or allogeneic serum at 20 or 95 % and DMSO at 10 or 5 %. *Stem Cell Res Ther* (2015) 6: doi: 10.1186/s13287-015-0230-y

58.	Paterson YZ, Rash N, Garvican ER, Paillot R, Guest DJ. Equine mesenchymal stromal cells and embryo-derived stem cells are immune privileged in vitro. *Stem Cell Res Ther* (2014) 5: doi: 10.1186/scrt479

59.	Koerner J, Nesic D, Romero JD, Brehm W, Mainil-Varlet P, Grogan SP. Equine Peripheral Blood-Derived Progenitors in Comparison to Bone Marrow-Derived Mesenchymal Stem Cells. *Stem Cells* (2006) 24:1613–1619. doi: 10.1634/stemcells.2005-0264

60.	Rodolfo Nino-Fong BPEGJCR-LWMLM. Development of a biologically immortalized equine stem cell line. *The Canadian Journal of Veterinary Research* (2021) 85:293–301.

61.	Ishikawa S, Horinouchi C, Murata D, Matsuzaki S, Misumi K, Iwamoto Y, Korosue K, Hobo S. Isolation and characterization of equine dental pulp stem cells derived from thoroughbred wolf teeth. *Journal of Veterinary Medical Science* (2017) 79:47–51. doi: 10.1292/jvms.16-0131

62.	Tasma Z HWDTSKKMGYHDHFHCCLW. Production of extracellular vesicles from equine embryo-derived mesenchymal stromal cells.. . *Reproduction* (2022) 164:143–154.

63.	Li H, Xiong S, Masieri FF, Monika S, Lethaus B, Savkovic V. Mesenchymal Stem Cells Isolated from Equine Hair Follicles Using a Method of Air-Liquid Interface. *Stem Cell Rev Rep* (2023) 19:2943–2956. doi: 10.1007/s12015-023-10619-w

64.	Yang Q, Lopez MJ. Ultrastructural morphology is distinct among primary progenitor cell isolates from normal, inflamed, and cryopreserved equine hoof tissue and CD105+K14+ progenitor cells. *In Vitro Cell Dev Biol Anim* (2019) 55:641–655. doi: 10.1007/s11626-019-00380-1

65.	Yang Q, Pinto VMR, Duan W, Paxton EE, Dessauer JH, Ryan W, Lopez MJ. In vitro characteristics of heterogeneous equine hoof progenitor cell isolates. *Front Bioeng Biotechnol* (2019) 7: doi: 10.3389/fbioe.2019.00155

66.	Adams MK, Goodrich LR, Rao S, Olea-Popelka F, Phillips N, Kisiday JD, McIlwraith CW. Equine bone marrow-derived mesenchymal stromal cells (BMDMSCs) from the ilium and sternum: Are there differences? *Equine Vet J* (2013) 45:372–375. doi: 10.1111/j.2042-3306.2012.00646.x

67.	Rink BE, Amilon KR, Esteves CL, French HM, Watson E, Aurich C, Donadeu FX. Isolation and characterization of equine endometrial mesenchymal stromal cells. *Stem Cell Res Ther* (2017) 8: doi: 10.1186/s13287-017-0616-0

68.	De Schauwer C, Meyer E, Van de Walle GR, Van Soom A. Markers of stemness in equine mesenchymal stem cells: A plea for uniformity. *Theriogenology* (2011) 75:1431–1443. doi: 10.1016/j.theriogenology.2010.11.008

69.	Iacono E, Brunori L, Pirrone A, Pagliaro PP, Ricci F, Tazzari PL, Merlo B. Isolation, characterization and differentiation of mesenchymal stem cells from amniotic fluid, umbilical cord blood and Wharton’s jelly in the horse. *Reproduction* (2012) 143:455–468. doi: 10.1530/REP-10-0408

70.	Fülber J, Maria DA, Da Silva LCLC, Massoco CO, Agreste F, Baccarin RYA. Comparative study of equine mesenchymal stem cells from healthy and injured synovial tissues: An in vitro assessment. *Stem Cell Res Ther* (2016) 7: doi: 10.1186/s13287-016-0294-3

71.	Hillmann A, Ahrberg AB, Brehm W, Heller S, Josten C, Paebst F, Burk J. Comparative characterization of human and equine mesenchymal stromal cells: A basis for translational studies in the equine model. *Cell Transplant* (2016) 25:109–124. doi: 10.3727/096368915X687822

72.	Reed SA, Johnson SE. Equine umbilical cord blood contains a population of stem cells that express Oct4 and differentiate into mesodermal and endodermal cell types. *J Cell Physiol* (2008) 215:329–336. doi: 10.1002/jcp.21312

73.	Punzón E, Salgüero R, Totusaus X, Mesa-Sánchez C, Badiella L, García-Castillo M, Pradera A. Equine umbilical cord mesenchymal stem cells demonstrate safety and efficacy in the treatment of canine osteoarthritis: a randomized placebo-controlled trial. *J Am Vet Med Assoc* (2022) 260:1947–1955. doi: 10.2460/javma.22.06.0237

74.	Murata D, Yamasaki A, Matsuzaki S, Sunaga T, Fujiki M, Tokunaga S, Misumi K. Characteristics and multipotency of equine dedifferentiated fat cells.

75.	McDuffee LA, Anderson GI. In Vitro Comparison of Equine Cancellous Bone Graft Donor Sites and Tibial Periosteum as Sources of Viable Osteoprogenitors. *Veterinary Surgery* (2003) 32:455–463. doi: 10.1053/jvet.2003.50060

76.	Laurie A. McDuffee GIAGMWDAJR. In vitro heterogeneity of osteogenic cell populations at various equine skeletal sites. *The Canadian Journal of Veterinary Research* (2006) 70:277–284.

77.	Storch C, Fuhrmann H, Schoeniger A. Hox gene expressions in cultured articular and nasal equine chondrocytes. *Animals* (2021) 11: doi: 10.3390/ani11092542

78.	Nixon AJ, Lust G, Vernier -Singer M. Isolation , propagation, and cryopreservation of equine articular chondrocytes.

79.	Rosser J, Bachmann B, Jordan C, Ribitsch I, Haltmayer E, Gueltekin S, Junttila S, Galik B, Gyenesei A, Haddadi B, et al. Microfluidic nutrient gradient–based three-dimensional chondrocyte culture-on-a-chip as an in vitro equine arthritis model. *Mater Today Bio* (2019) 4: doi: 10.1016/j.mtbio.2019.100023

80.	Gurusinghe S, Hilbert B, Trope G, Wang L, Bandara N, Strappe P. Generation of Immortalized Equine Chondrocytes With Inducible Sox9 Expression Allows Control of Hypertrophic Differentiation. *J Cell Biochem* (2017) 118:1201–1215. doi: 10.1002/jcb.25773

81.	Daniela Ennulat Scott A. Brown. CANINE AND EQUINE MESANGIAL CELLS IN VITRO. *In Vitro Cell Dev Biol--Animal* (1995) 31:574–578.

82.	Anderson LW, Banks KL. Collection and Cultivation in Vitro of Equine Mammary Macrophages.

83.	Spaas JH CKBLBCV de WGR. Stem/Progenitor Cells in Non-Lactating Versus Lactating Equine Mammary Gland. *Stem Cells Dev* (2012) 21:3055–3067.

84.	McClellan A, Paterson YZ, Paillot R, Guest DJ. Equine Fetal, Adult, and Embryonic Stem Cell-Derived Tenocytes Are All Immune Privileged but Exhibit Different Immune Suppressive Properties in Vitro. *Stem Cells Dev* (2019) 28:1413–1423. doi: 10.1089/scd.2019.0120

85.	Banks KL, Greenlee A. Isolation and Identification of Equine Lymphocytes and Monocytes.

86.	Kadoi K, Lelli R, Caporale V, Onodera T. Establishment of equine and murine monocytic cell lines and their susceptibility to West Nile virus. (2012). 22–026 p. http://www.interesjournals.org/IRJBB

87.	Fidalgo-Carvalho I, Craigo JK, Barnes S, Costa-Ramos C, Montelaro RC. Characterization of an equine macrophage cell line: Application to studies of EIAV infection. *Vet Microbiol* (2009) 136:8–19. doi: 10.1016/j.vetmic.2008.10.010

88.	Ziegler A, Everett H, Hamza E, Garbani M, Gerber V, Marti E, Steinbach F. Equine dendritic cells generated with horse serum have enhanced functionality in comparison to dendritic cells generated with fetal bovine serum. *BMC Vet Res* (2016) 12: doi: 10.1186/s12917-016-0880-8

89.	Sharon Witonsky DPRMGJDVB-MDMSAADP. Immunologic analysis of blood samples obtained from horses and stored for twenty-four hours. *AJVR* (2003) 64:

90.	Laurie A. McDuffee. Comparison of isolation and expansion techniques for equine osteogenic progenitor cells from periosteal tissue. *The Canadian Journal of Veterinary Research* (2012) 76:91–98.

91.	Rose B V., Cabrera-Sharp V, Firth MJ, Barrelet FE, Bate S, Cameron IJ, Crabtree JR, Crowhurst J, McGladdery AJ, Neal H, et al. A method for isolating and culturing placental cells from failed early equine pregnancies. *Placenta* (2016) 38:107–111. doi: 10.1016/j.placenta.2015.12.014

92.	Mahera JK, Tresnanb DB, Deacon S, Hannah L, Antczak DF, The ", Baker JA. Analysis of MHC Class I Expression in Equine Trophoblast Cells Using In Situ Hybridization. (1996). 351–359 p.

93.	Thway TM, Clay CM, Maher JK, Reed DK, Mcdowell KJ, Antczak DF, Eckert RL, Nilson JH, Wolfe MW. Immortalization of equine trophoblast cell lines of chorionic girdle cell lineage by simian virus-40 large T antigen. http://www.endocrinology.org

94.	Deboer ML, Martinson KM, Pampusch MS, Hansen AM, Wells SM, Ward C, Hathaway M. Cultured equine satellite cells as a model system to assess leucine stimulated protein synthesis in horse muscle. *J Anim Sci* (2018) 96:143–153. doi: 10.1093/jas/skx028

95.	Naylor Bvetmed RJ, Piercy Vetmb RJ. Development of a clonal equine myoblast cell line capable of terminal differentiation into mature myotubes in vitro. (2015).

96.	Rooney MF, Neto NGB, Monaghan MG, Hill EW, Porter RK. Conditionally immortalised equine skeletal muscle cell lines for in vitro analysis. *Biochem Biophys Rep* (2023) 33: doi: 10.1016/j.bbrep.2022.101391

97.	Dwayne H. Rodgerson DMJKBDPGLFDLK. Characterization of cultured smooth muscle cells obtained from the palmar digital arteries of horses. *AJVR* (2000) 61:

98.	Lynch TM, Caron JP, Arnoczky SP, Lloyd JW, Stick JA, Render JA. Influence of exogenous hyaluronan on synthesis of hyaluronan and collagenase by equine synoviocytes.

99.	Beaumont RE, Smith EJ, David C, Paterson YZ, Faull E, Guest DJ. Equine adult, fetal and ESC-tenocytes have differential migratory, proliferative and gene expression responses to factors upregulated in the injured tendon. *Cells and Development* (2025) 181: doi: 10.1016/j.cdev.2025.204003

100.	Pechanec MY, Boyd TN, Baar K, Mienaltowski MJ. Adding exogenous biglycan or decorin improves tendon formation for equine peritenon and tendon proper cells in vitro. *BMC Musculoskelet Disord* (2020) 21: doi: 10.1186/s12891-020-03650-2

101.	Baldwin DM RJMMLBDN. Direct effects of free and conjugated steroids on GnRH stimulated LH release in cultured equine anterior pituitary cells. . *Journal of Reproduction and fertility Supplement 1991 ;44:327-332*
